# A Primer on Repeated Sitting Exposure and the Cardiovascular System: Considerations for Study Design, Analysis, Interpretation, and Translation

**DOI:** 10.3389/fcvm.2021.716938

**Published:** 2021-08-17

**Authors:** Lee Stoner, Bethany Barone Gibbs, Michelle L. Meyer, Simon Fryer, Daniel Credeur, Craig Paterson, Keeron Stone, Erik D. Hanson, Robert J. Kowalsky, Masahiro Horiuchi, Christopher P. Mack, Gaurav Dave

**Affiliations:** ^1^Department of Exercise and Sport Science, University of North Carolina at Chapel Hill, Chapel Hill, NC, United States; ^2^Department of Health and Human Development and Clinical and Translational Science, University of Pittsburgh, Pittsburgh, PA, United States; ^3^Department of Emergency Medicine, University of North Carolina at Chapel Hill, Chapel Hill, NC, United States; ^4^School of Sport and Exercise, University of Gloucestershire, Gloucester, United Kingdom; ^5^Department of Biology, Ave Maria University, Ave Maria, FL, United States; ^6^Department of Health and Kinesiology, Texas A&M University-Kingsville, Kingsville, TX, United States; ^7^Division of Human Environmental Science, Mount Fuji Research Institute, Yamanashi, Japan; ^8^Department of Pathology and Laboratory Medicine, University of North Carolina at Chapel Hill, Chapel Hill, NC, United States; ^9^Division of General Medicine and Clinical Epidemiology, University of North Carolina at Chapel Hill, Chapel Hill, NC, United States

**Keywords:** biological plausibility, ecological validity, external validity, internal validity, methodology, sedentary behavior, arterial stiffness, endothelial function

## Abstract

Sedentary behavior, particularly sitting, is ubiquitous in many contemporary societies. This is a major societal concern considering the evidence for a strong association between sitting behavior and cardiovascular disease morbidity and mortality. Unsurprisingly, leading public health agencies have begun to advocate “reduction” in sitting behavior. Though, the guidelines are typically vague and non-specific. The lack of specific guidelines for prolonged sitting is attributable to the absence of available evidence to facilitate guideline development. To inform policy, well-designed randomized controlled trials are required to test the efficacy of specific and translatable sitting reduction strategies. To guide the design of randomized controlled trials, this review postulates that several gaps in the literature first need to be filled. Following a general discussion of the importance of sitting behavior to contemporary societies, each of the following are discussed: (i) acute sitting exposure and systems physiology; (ii) recommendations for a systems physiology toolbox; (iii) study design considerations for acute sitting exposure; and (iv) translation of sitting-focused research.

## Introduction

**Figure 1:**
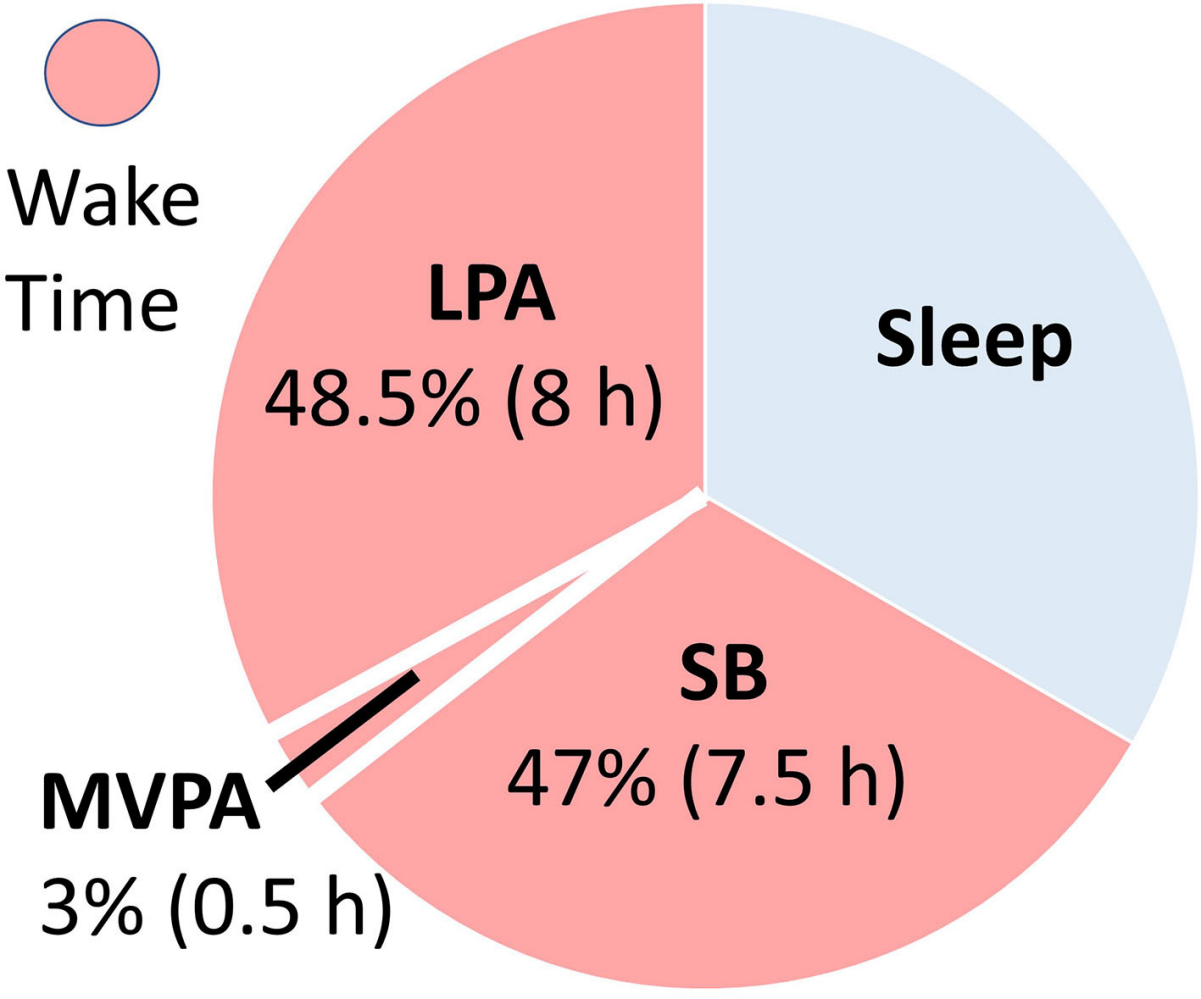
Example of a typical 24-h activity cycle for individuals in modernized economies. The average adult engages in sedentary behaviors (SB) ~half their waking hours, with the majority of the remaining waking hours engaged in light physical activity (LPA). Time spent in moderate-to-vigorous physical activity (MVPA) is typically minimal (~3% of the day), even for adults meeting recommended levels.

**Figure 2:**
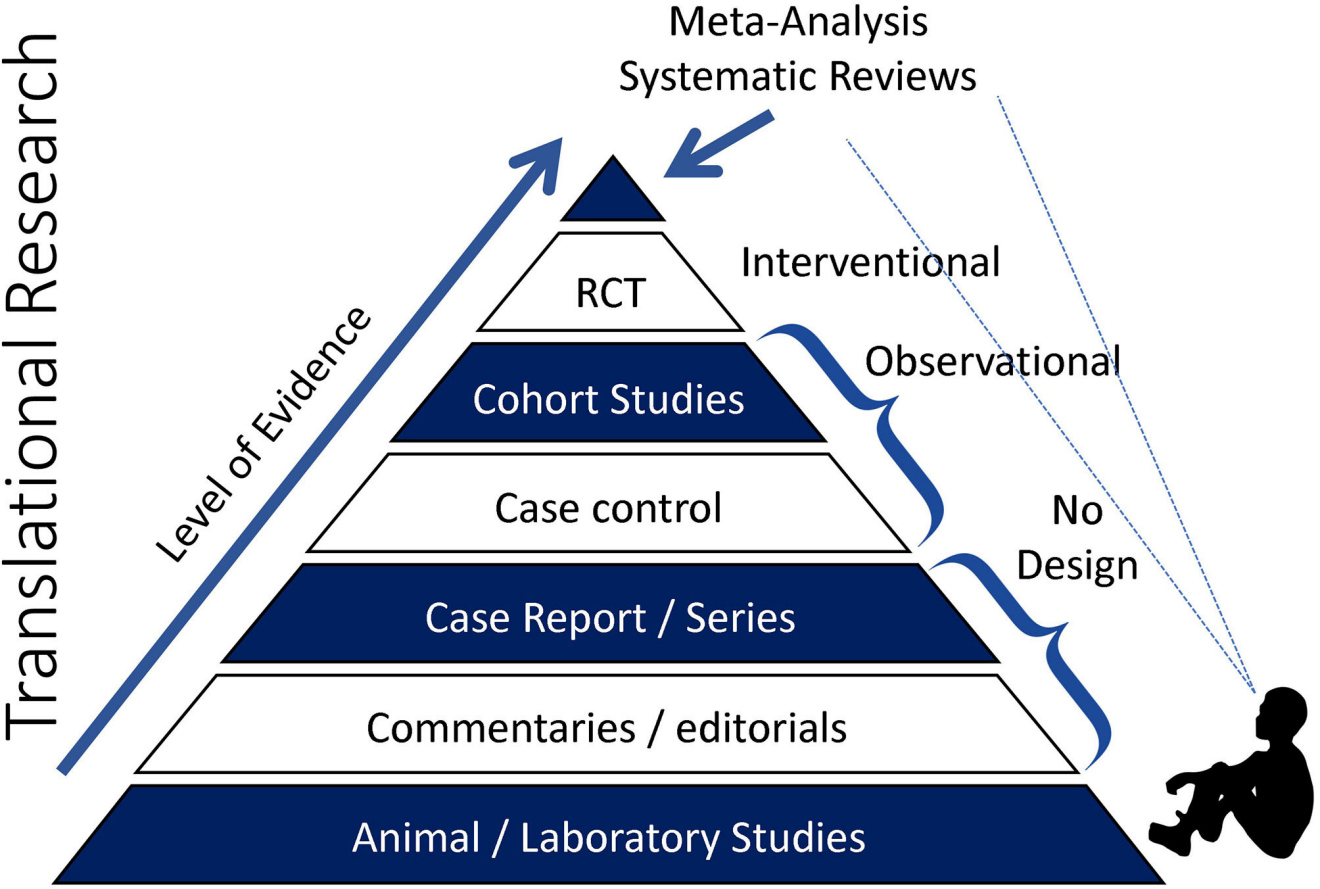
Pyramid of clinical evidence. With respect to sedentary behavior-focused research and the capacity to develop sedentary behavior-reduction policy, we are currently sitting somewhere near the bottom of the pyramid peering up.

Sedentary behavior (SB), particularly sitting, is ubiquitous in many contemporary societies (Figure 1) (1). This is a major societal concern in light of the evidence for a strong association between SB and cardiovascular disease (CVD) mortality. However, while leading public health agencies, including the World Health Organization (WHO), have begun to advocate “reduction” in SB, the guidelines are typically vague and non-specific (2, 3). To inform SB policy, well-designed cohort studies and randomized controlled trials (RCTs) are needed (see Figure 2) to test the efficacy of specific and translatable SB reduction strategies. To assist in the design of RCTs, this review postulates that several gaps in the literature first need to be filled. Following a general discussion of the importance of SB to contemporary societies, each of the following are discussed:

- Acute SB exposure and systems physiology, i.e., how and why SB increases CVD risk.
- Systems physiology toolbox, i.e., methods to investigate the effects of acute SB exposure on systems physiology. The flow-mediated dilation (FMD) and pulse wave velocity (PWV) test are used as exemplars to provide context to the key considerations that need to be made.
- Study design consideration, i.e., how to design an acute SB study with forethought to internal validity, external validity, and ecological validity. The FMD and PWV tests are further used to provide context.
- Translation, i.e., steps to increase the likelihood of SB-focused research informing policy.

While some basic context is provided, it is not the intention of this review to comprehensively discuss the historical and socio-cultural considerations, or to comprehensively discuss potential mechanistic pathways. For clarity, the intention of this review is to provide a primer for important considerations to assist in the design of acute SB studies. Table 1 presents a glossary of terms, provided to assist in the interpretation of concepts specific to the sitting literature and discussed within this review. This review should be viewed as an addition to existing research design guidelines, including the Consolidated Standards of Reporting Trials (CONSORT) (4).

**Table 1:** Glossary of terms.

**Term**	**Definition**
24-h activity cycle	Activities conducted over a 24-h period including, sleep, sedentary behavior, light-intensity physical activity, and moderate-to-vigorous physical activity.
Accuracy (Validity)	Deviation of a measure from its true value, including systematic (constant bias) and random error (variable) components.
Arterial stiffness	Fundamental mechanical behavior or rigidity of the material properties of the artery wall, determined by both structural and functional components.
Biological plausibility	Proposal of a causal association (or relationship between a cause and outcome) that is consistent with existing biological and medical knowledge.
Cardiovascular disease (CVD)	A general term for pathologies affecting the heart or blood vessels, including cerebrovascular disease, myocardial infarction, coronary heart disease, and peripheral artery disease.
Exercise	A subset of physical activity that is planned, structured, and repetitive.
Ecological validity	The degree to which results obtained from research or experimentation are representative of conditions in the wider world.
Endothelial function	The ability of the endothelium to adequately perform its physiological roles in maintaining homeostasis.
External validity	The degree to which causal relationships can be generalized to different measures, persons, settings, and times.
Intermediate outcome	A surrogate measure (e.g., pulse wave velocity) used to measure the effect of a treatment that may correlated with a real clinical endpoint (e.g., cardiovascular disease).
Internal validity	Degree to which a study establishes the cause-and-effect relationship between the treatment and the observed outcome.
Metabolic equivalent (MET)	Unit used to describe the absolute intensity of physical activity. A ratio of your working metabolic rate relative to your resting metabolic rate.
Physical activity	Any bodily movement produced by skeletal muscles that requires energy expenditure.
Physical inactivity	An insufficient level of moderate-vigorous physical activity level to meet present physical activity recommendations.
Policy	A course or principle of action adopted or proposed by an organization or individual.
Precision (Reliability)	Dispersion of a measurement or degree to which a measure contained on one occasion is repeated on a second occasion, including intra-rater (test-retest) and inter-rater components.
Randomized controlled trial (RCT)	People are randomly assigned to two (or more) groups, typically including one control and one or more experimental groups.
Sedentary behavior (SB)	Any waking behavior characterized by an energy expenditure ≤ 1.5 METs, while in a sitting, reclining or lying posture.
Translation	The use of scientific evidence by decision makers to inform and generating health policy.

## Importance of Sedentary Behavior to Society

Since the mid-20th century, societal changes have led to increased SB, particularly sitting (5). Today, the average American spends 55% (7.7-h/day) of their waking time sedentary, with much of that time spent sitting, and inactive (1). The terms “physical inactivity” and “sedentary behavior” have been used interchangeably. However, physical inactivity refers to not meeting moderate-to-vigorous physical activity (MVPA) guidelines, and SB refers to very low intensity behaviors ( ≤ 1.5 metabolic equivalents) in a seated, reclined, or supine posture (6). SB is a distinct CVD risk factor (1, 7), and interrupting SB with light intensity physical activity has health benefits independent from MVPA (8, 9). Despite SB being a large part of our daily behavior (see Figure 1), public health programs and policies have primarily focused on other modifiable health behaviors, such as diet, smoking, and MVPA. Little is known about optimal prescription for reducing SB (2).

Using MVPA as an exemplar, the 2018 Physical Activity Guidelines provide a clear public health message: “For substantial health benefits, adults should do at least 150 min per week…of moderate-intensity…aerobic physical activity” (2). These guidelines, which are based on the FITT (frequency, intensity, time, and type) principle, are driven by numerous RCTs and subsequent meta-analyses exploring the health benefits of physical activity (2). Such RCTs and meta-analysis data are unavailable for SB. However, it should be acknowledged that, while the first physical activity study was published in 1953 (10), SB-focused research is in its infancy. As such, the 2018 Physical Activity Guidelines do recommend sitting less, based on the “strong” overall and dose-response association between SB and CVD mortality, but they do not provide specific guidelines for sitting substitution (2). The absence of specific guidelines can be attributed to the fact that very few studies, mostly cross-sectional, have investigated the physiological mechanisms of SB and its association with CVD. This incomplete mechanistic understanding limits the ability to identify informed sitting-interruption strategies. The next part of this review will briefly discuss plausible physiological mechanisms, specifically as they relate to sitting behavior, followed by an overview of available tools to investigate these mechanisms.

## Systems Physiology and Acute Exposure to Prolonged Sitting

**Figure 3:**
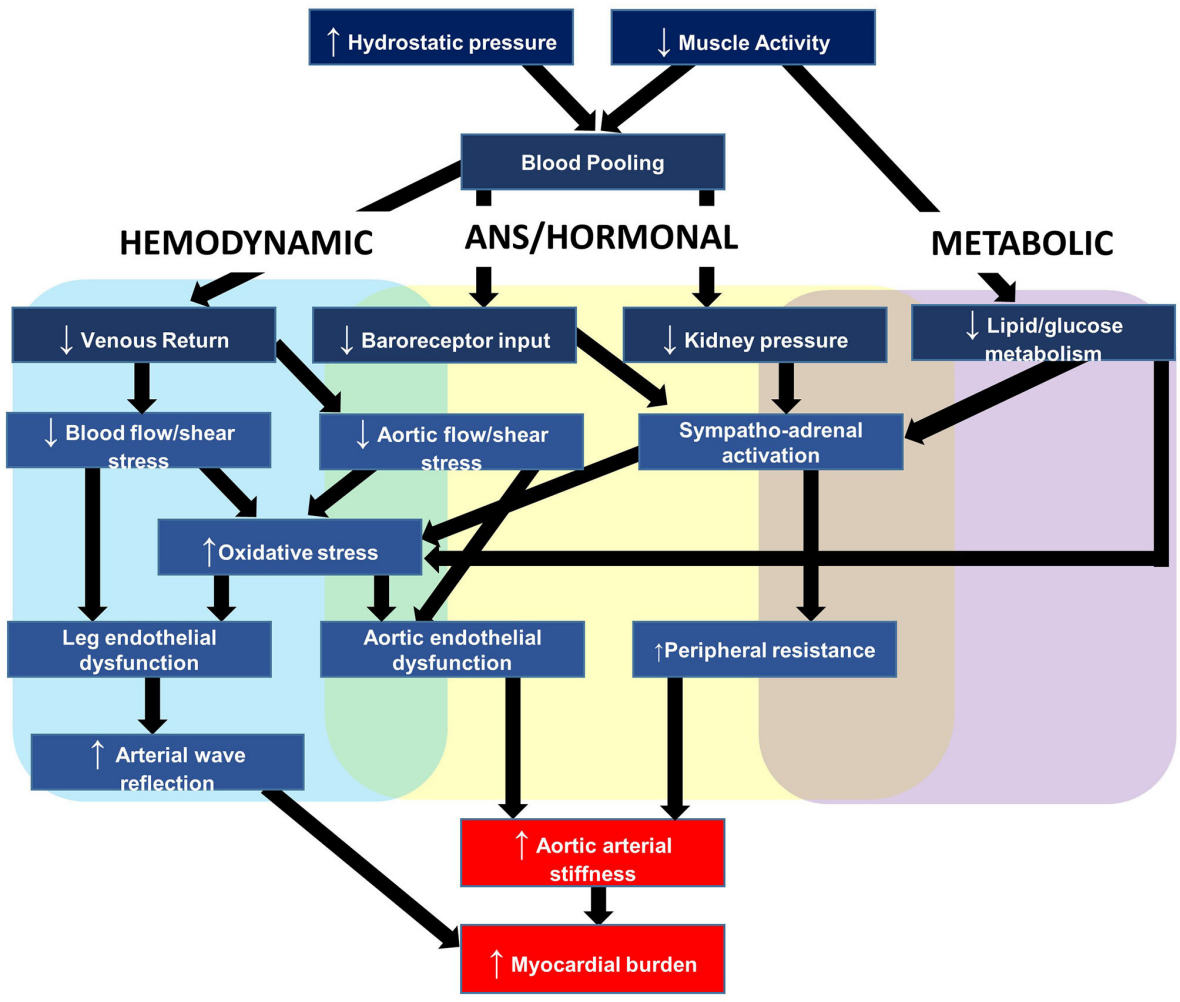
Hypothesized mechanisms linking repeated prolonged sedentary behavior (sitting) exposure to cardiovascular disease.

To provide context to the sections that follow, this section briefly outlines a plausible working model for the relationship between acute prolonged sitting exposure and negative cardiovascular responses. The primary outcome of this model, depicted in Figure 3, is aortic arterial stiffness, a measure explained in further detail below and that we have reported to increase in response to uninterrupted prolonged sitting (11–14). The elastic aorta is directly proximal to the heart and is responsible for dampening the speed and amplitude of retrograde pressure waves that increase the heart's workload during systole (myocardial burden) (15). This measure is particularly appealing because arterial stiffness is a multifactorial measure of central hemodynamics, dependent on blood pressure and intrinsic arterial structural and functional properties (15). Therefore, an increase in aortic arterial stiffness not only signifies increased myocardial burden and target organ damage susceptibility, but also reflects the stress being placed on the aorta and the potential for detrimental changes to the vessel.

Our working hypothesis is that acute sitting-induced increases in arterial stiffness are likely driven by local hemodynamic changes and compounded by a number of detrimental autonomic, hormonal, and metabolic factors. With respect to hemodynamics, we have reported that hydrostatic pressure and lack of muscle pump activity led to lower extremity venous blood pooling, the slower transit time of blood through the venous circulation, which decreased venous return and was inversely associated with stroke volume (16). This decreased stroke volume in turn reduces shear stress (17, 18), the primary regulator of endothelial function (19). Decreased shear stress promotes oxidative stress, endothelial dysfunction, and acutely increases aortic arterial stiffness (20–24). As mentioned above, the increased aortic stiffness may exacerbate myocardial burden as a function of the increased speed and amplitude of retrograde pressure waves. Changes to peripheral vessels, particularly in the lower-limbs, may compound the retrograde pressure waves. In support, we reported endothelial dysfunction (25) and increased arterial stiffness in the legs (26). Lower-limb leg endothelial dysfunction is induced by the pro-atherogenic shear stress profile as a result of decreased stroke volume and alterations in vascular resistance near tortuous vessels (27–29).

Autonomic and hormonal changes further elevate aortic arterial stiffness by increasing systemic vascular resistance (30). The autonomic changes are driven by reduced pressure along the arterial baroreceptors, and the hormonal changes by reduced blood pressure along the kidneys, which stimulates the renin-angiotensin aldosterone system (31). Metabolically, lack of muscle contractile stimulation suppresses lipoprotein lipase activity—required for triglyceride uptake and high-density lipoprotein production—and glucose uptake (32, 33). Elevated plasma glucose promotes higher circulating insulin concentrations, which contribute to arterial stiffness *via* a combination of enothelin-1 release from mitogen-activated protein kinase activation, and centrally-mediated adrenergic vasoconstriction (34).

The hemodynamic, autonomic, hormonal, and metabolic changes briefly described in this section lead to acutely increased aortic arterial stiffness. However, we hypothesize that repeated increases in arterial stiffness leads to structural remodeling of vessel walls (15), and this helps to explain, in part, the positive association between chronic SB and aortic arterial stiffness (35), and between SB and CVD (2, 3).

## Sitting Toolbox

Table 2 outlines example tools available to acute sitting-focused researchers and relates these tools to the working model outlined in Figure 3. At the end of this section, we provide further details of two commonly used tools, FMD and PWV, which will subsequently be further discussed in terms of internal validity in the proceeding section Internal Validity Considerations. As outlined in Table 3, when selecting appropriate tools, a number of considerations need to be made, starting with “what is your research question?” Is the purpose to elucidate the mechanisms by which acute prolonged sitting exposure leads to increased myocardial burden? If so, which tool(s) can reflect the myocardial burden, and which physiological systems are contributing to this burden? Additionally, are these physiological changes systemic, such as hormonal changes, or are the physiological changes more local, such as increased arterial stiffness in the lower extremities? If the physiological changes are more local, what caused said physiological changes and how do these local changes influence systems physiology? For example, what mechanisms contribute to decreased endothelial function in the lower extremities, and how does this endothelial function contribute to myocardial burden. Answering these questions requires appropriate tool selection.

**Table 2:** Research toolbox for investigating the effect of acute sitting exposure on the cardiovascular system.

**Outcome**			**Cost**		**Test**			**Rationale**
**Construct**	**Measure**	**Technology**	**Instrument**	**Consumable**	**Time (min)**	**Skill**	**Reliability**	
**Hemodynamic**
Arterial stiffness	PWV	Oscillometry or tonometry	>$15 k	Negligible	5	Med	High	Reflects stress placed on arterial wall.
BP (Peripheral)	Peripheral	Oscillometry	< $1 k	Negligible	5	Low	High	Force exerted on a vessel.
BP (Central)	PWA	Oscillometry	>$15 k	Negligible	5	Low	High	Reflects myocardial load.
Blood flow	i)Aorta	i)CW-US	>$40 k	Negligible	<5	High	Med	↓blood flow-induced shear stress induces endothelial dysfunction.
	ii)Brain	ii)TCD						
	iii)Peripheral	iii)Doppler US						
Blood pooling	Deoxy[heme]	NIRS	>$10 k	Negligible	5	Med	High	Reflects blood pooled in venous compartment.
Endothelial Function	FMD	US	>$40 k	Negligible	10	High	Med	Represents macrovascular dysfunction.
Microvascular Function	Reactive hyperemia	US	>$40 k	Negligible	8	High	Med	Represents microvascular dysfunction
SVR	CO/ MAP	Finger PPG	>$30 k	Negligible	1	Low	High	Moderates blood pooling and venous return.
Wave reflection	PWA	Oscillometry	>$15 k	Negligible	5	Low	High	Wave reflection increases myocardial load.
**Autonomic**
PNS	HRV	ECG	>$10 k	Negligible	5	Low	High	Reflects change in PNS activity.
SNS	Catecholamines	ELISA		$500/kit	5	Med	High	Reflects change in SNS activity.
**Hormonal**
CACs	Venous blood	Flow cytometry	>$20 k	$65/person	5	Med	High	Reflect endothelial repair.
EMPs	Venous blood	Flow cytometry	>$20 k	$50/person	5	Med	High	Reflect endothelial damage.
Insulin	Venous blood	ELISA		$500/kit	5	Med	High	May ↑ SNS activity.
RAAS	Venous blood	ELISA		$500/kit	5	Med	High	↑BP.
**Metabolic**
Glucose	Venous blood	ELISA		$500/kit	5	Med	High	Reflects glycemic control.
Lipids	Venous blood	ELISA		$500/kit	5	Med	High	May impair vascular function.

*BP, blood pressure; CACs, circulating angiogenic cells; CO, cardiac output; CVD, cardiovascular disease; CW-US, continuous-wave ultrasound; deoxy[heme], deoxygenated heme concentration; ECG, electrocardiogram; ELISA, enzyme-linked immunosorbent assay; EMPs, endothelial micro-particles; FMD, flow-mediated dilation; HRV, heart rate variability; MAP, mean arterial pressure; NIRS, near infrared spectroscopy; PNS, parasympathetic nervous system; PPG, photo-plethysmography; PWA, pulse wave analysis; PWV, pulse wave velocity; RAAS, renin-angiotensin aldosterone system; SB, sedentary behavior; SNS, sympathetic nervous system; SVR, systemic vascular resistance; TCD, transcranial Doppler; US, ultrasound*.

**Table 3:** Measurement considerations for studies investigating the effect of acute sitting exposure on the cardiovascular system.

**Consideration**	**Example(s)**	**Recommendation(s)**
**Physiological**		
Research question	Which physiological systems are relevant to the question?	Ensure question is clear and addressable.
Multi-system	Prolonged sitting impacts multi systems, how can these changes be accounted for?	Do you need to control or monitor for potential confounders?
Central vs. local	Will local physiological changes impact systems physiology?	Consider interactions between local and systems physiology and how this can be controlled/measured.
Stimulus time	What duration needed for desired physiological response?	Ensure prolonged sitting bout is sufficient for inducing desired physiology change.
**Toolbox selection**		
Research question	Will the tools answer your question?	Will the tools measure change in the desired construct?
Level of invasiveness	Will invasive measurements arouse the participant?	Limit measurement of disruptive/invasive measurements.
Subject burden	Will repeated measurements arouse the participant?	Limit measures to those pertinent to research question.
Measurement time	How long will each measurement take?	Consider subject burden and impact of measurement on subsequent measures.
Measurement frequency	Too frequent measurement may act as a stimulus.	Limit measure frequency to minimize potential carry-over effect.
Measurement posture	Are the measurements validated for use in the measurement posture?	Measure under recommended/standardized conditions.
**Methodological**		
Study design	Parallel group or randomized cross-over?	Provide a rationale for design choice.
Single vs. multi-day	Time needed for physiological response?	Determine whether a single sitting bout is sufficient to address question.
Randomization process	Which process, and who does it?	Third party, blinded to testing conditions, should complete using established software.
Blinding	Should/can the participant and operator be blinded?	Blind subjects, researchers, and statisticians where feasible.
Adherence to protocol	Will poor adherence reduce the treatment impact?	Measure and report protocol adherence. Report missing data.
Power calculations	Is the study powered to address the question with respect to the primary outcome (under proposed testing condition, and specific to lab/observer)?	Should be pre-planned. Clearly describe calculations.
Statistical	Address dropouts, confounders, and clinical inference.	Describe statistical procedures in sufficient detail to replicate.
**Internal validity**		
Regression	If subjects are selected on basis of extreme scores, will the distribution regress to mean with repeated testing?	Describe rationale for population selection.
Treatment randomization	How will the intervention be given from one participant to the next?	Randomize treatment allocation
Selection	What should be the appropriate control group condition?	The control group/condition should be appropriately matched to the experimental group/condition.
History	Is there a carry-over or period effect?	Provide rationale for wash-out period and statistically test.
Drop-outs	Are those who remain different?	Consider intention-to-treat framework or decide strategy for drop-outs *a priori*.
Pre-assessment control	Consider physical activity and sleep, caffeine, medications and supplements?	Control and standardize as much as possible. Consider food diaries, accelerometers and other tools for monitoring.
During assessment control	Consider environment (temperature, humidity, noise), meal consumption, fluid replacement, and fidgeting?	Standardize and control environmental conditions.
Instrumentation	Consider precision, accuracy, single-observer, measurement posture, signal interpretation, and covariates?	Ensure choice of instrumentation is appropriate for research question(s), technicians, participants, and protocols.
**External validity**		
Internal validity	Will internal validity compromise external validity?	Balance robust methods with the need for generalizability.
Sampling	Random sampling and allocation?	Provide clear inclusion/exclusion criteria in the methods.
Sex	Control for menstrual cycle?	Consider whether changes in the menstrual cycle are relevant to your research question(s) and whether participants need to be in a particular phase of the cycle or whether this limits generalizability.
Race	Will race moderate the effect of prolonged sitting on the outcomes?	Provide clear rationale for race stratification and statistical analysis.
Age	Will the same sitting interruption strategies work for young and older populations?	Consider feasibility of strategy in older age groups.
Clinical and Special Populations	Will the findings generalize to clinical (e.g., Type II diabetes) and special (e.g., spinal cord injury) population?	Consider whether tested mechanism(s) or sitting reduction strategies need to be adjusted.
**Ecological validity**		
Feasibility	Will people do the sitting interruption strategy?	Consider the balance between mechanistically optimal vs. feasible sitting interruption strategies.
Time of day	Will testing in the morning (to standardize) conditions limit the findings?	Consider whether the effects of sitting on body systems differ across the day.
Interaction with other behaviors	What other behaviors do people engage while sitting?	Consider whether co-occurrence of lifestyle behaviors impact sitting response

Once viable tools have been identified, additional questions need to be asked. For example, how frequently do the measurements need to be made to capture the anticipated physiological response? If the frequency of measurements is too high, and/or the measurements are too disruptive to the subject, the subsequent increase in subject arousal may influence the physiological systems of interest. That is, the measurements themselves may confound internal validity (further discussion in Internal Validity Considerations.). For some measurements, this is not a concern, as they can be continuously captured without burdening the subject. For other measurements, such as blood pressure derived using oscillometry, there will be a burden to the subject. Ideally, measurements which are potentially disruptive to the subject are made at baseline and then at the end of the experiment. However, this brings an additional consideration: which posture should the measurements made in? Some of the measurements listed in Table 2 have been validated for use in the supine posture only and may be less precise in the seated posture. Measurement posture will be further considered in section Methodological Considerations.

### Endothelial Function: Flow-Mediated Dilation

**Figure 4:**
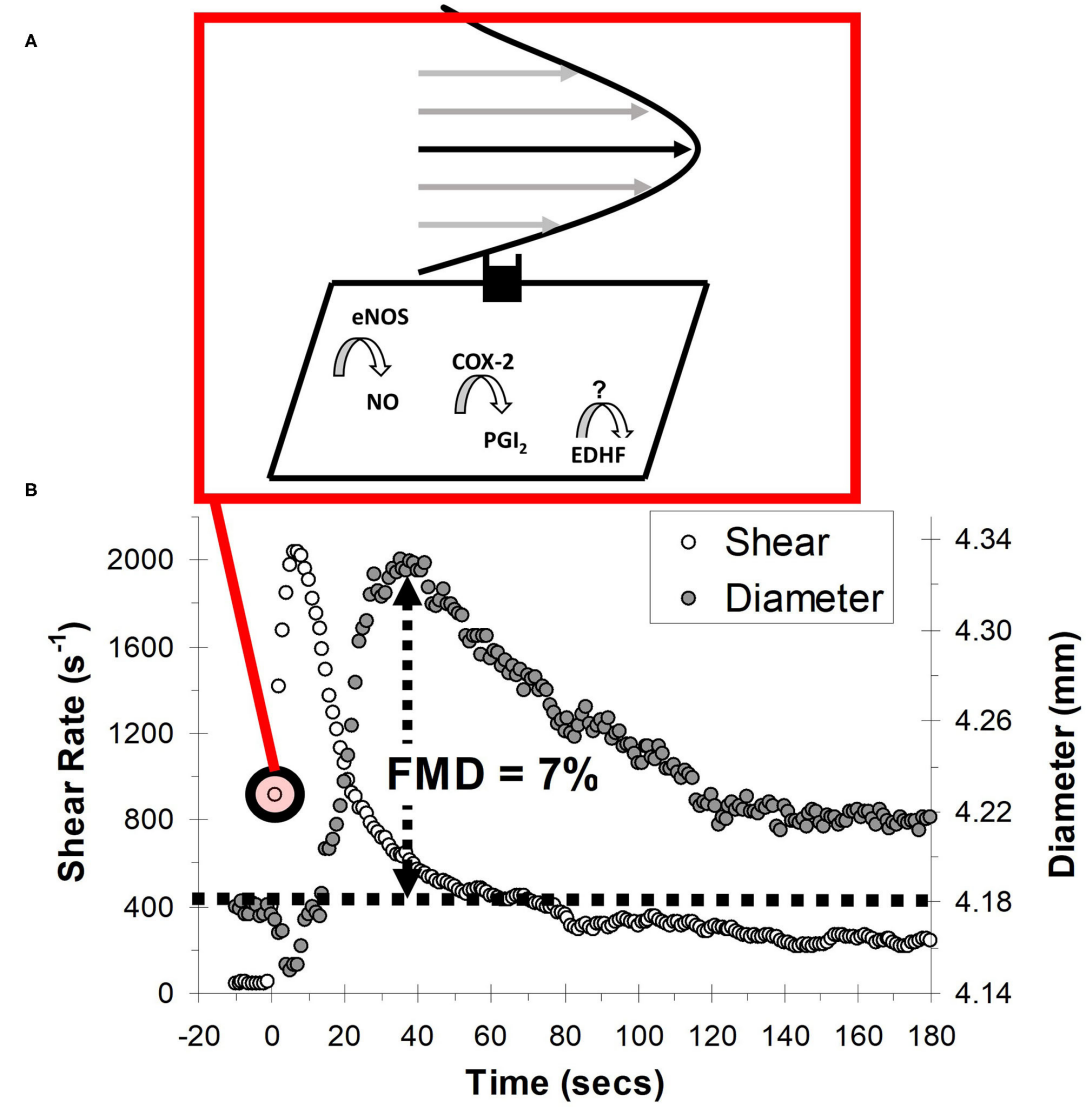
Schematic of the flow-mediated dilation (FMD) test. **(A)** Shear stress created by red blood cells flowing past the vessel wall is detected by mechanoreceptors in the endothelium, triggering a signal cascade whereby vasodilators, nitric oxide (NO) prostacyclin (PGI_2_) and endothelial hyperpolarizing factor (EDHF) are produced. Vasodilators subsequently diffuse to the vascular smooth muscle cells, prompting vasodilation. **(B)** Vessel diameter and shear rate responses before and after 5-min ischaemic stimulus. eNOS, endothelial nitric oxide synthase; COX-2, cyclooxygenase-2.

Endothelial dysfunction is a pivotal, yet potentially reversible step that precedes and predicts overt CVD (36). This is because the vascular endothelium is responsible for governing several aspects of vascular homeostasis, including regulating lipoprotein permeability, platelet aggregation, and vascular tone (37). A popular non-invasive test of endothelial function is FMD, which is quantified as the vasodilatory response to transiently increased shear stress during reactive hyperemia (Figure 4) (38). The vasodilatory (expressed as the percentage change in vessel diameter) and shear stress response can be measured using commercial duplex Doppler ultrasound coupled with dedicated image analysis software (e.g., FMD Studio, QUIPU; or Vascular Tools, Medical Imaging Applications). An important consideration, and one that will be expanded upon in the Internal Validity Considerations: Flow-Mediated Dilation section, is that the FMD test was validated for use in the brachial artery, and the vasodilatory responsiveness of this artery is thought to be a barometer of systemic endothelial function (38, 39).

Meta-analytic findings indicate that, after adjusting for confounding risk factors, a chronic increase of 1% (e.g., improving FMD from 6 to 7%) in brachial artery FMD equates to a 13% (95% confidence interval [CI]: 9–17%) reduction in the risk of future cardiovascular events (39). Our group recently conducted a meta-analysis (22 trials, *n* = 269) to summarize the effect of acute uninterrupted sitting exposure on FMD% (25). Uninterrupted prolonged sitting acutely decreased FMD% (weighted mead difference [WMD] = −2.14 %, 95% CI: −2.69 to −1.59), and sub-group analysis revealed that uninterrupted decreased lower- but not upper-limb FMD%. Additionally, we found that sitting interruption increased lower-extremity FMD% (beneficial) compared to uninterrupted sitting (WMD = 1.91%, 95% CI: 0.40–3.42). Subgroup analysis revealed a moderate but non-significant effect for aerobic interventions (WMD = 2.17, 95% CI: −0.34 to 4.67) and simple resistance activities (WMD = 2.40, 95% CI: −0.08 to 4.88) and a trivial effect for standing interruptions (WMD = 0.24, 95% CI: −0.90 to 1.38). In Internal Validity Considerations: Flow-Mediated Dilation section we will explore the internal validity of these findings.

### Arterial Stiffness: Pulse-Wave Velocity

**Figure 5:**
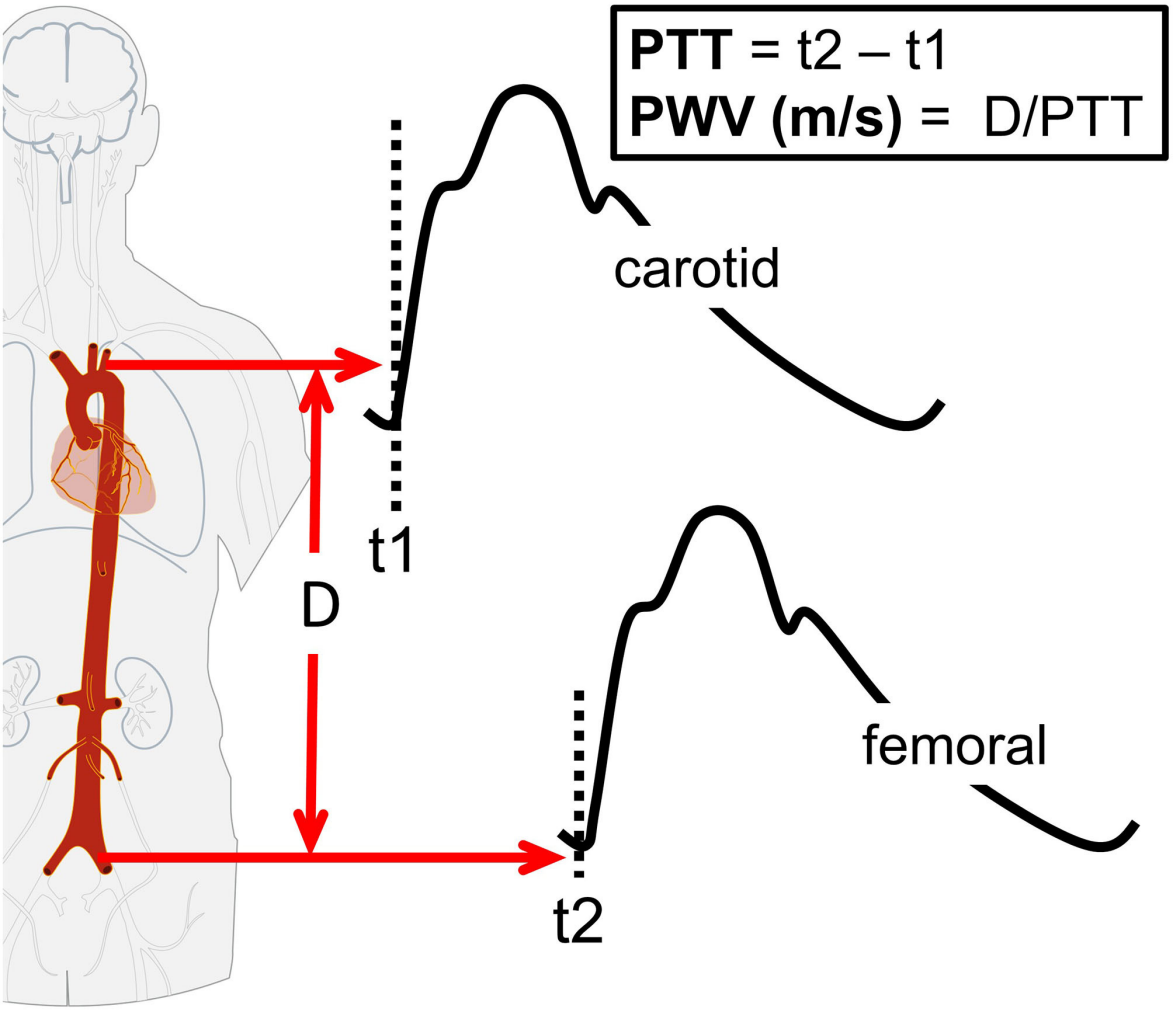
Schematic of carotid-femoral pulse-wave velocity (cfPWV). The cfPWV is calculated by dividing path length (D) by pulse transit time (PTT). The PTT is estimated as the difference in time (*t*) between the proximal (carotid artery) pressure waveform (*t*1) and distal (femoral artery) pressure waveform (*t*2).

Arterial stiffness is dependent on the functional and structural characteristics of a vessel (15, 22, 23). Functionally, acute changes in endothelial function influence arterial stiffness (22, 23). Structurally, arterial stiffness is dependent on the vessel wall extracellular matrix, including elastin and collagen (15). Decreased endothelial function and structural remodeling adversely affect pulsatile hemodynamics, leading to increased pulsatile stress toward end-organs, including the brain (40, 41), and greater arterial wave reflection with a resultant increase in myocardial load (15). A number of approaches are available for measuring arterial stiffness, of which the most widely used is the PWV between the carotid and femoral arteries (cfPWV, Figure 5). PWV is the speed at which the forward pressure wave is transmitted between two arterial segments, with faster PWV indicative of increased arterial stiffness. The PWV is traditionally assessed using tonometry, though more recently oscillometric-based devices have simplified the measurement procedure. Commercial oscillometric-based devices automate PWV calculations, confer excellent reliability (intra-class correlation coefficient [ICC]: 0.98), and compare the outcome to known reference values (Figure 5) (42).

A meta-analysis of 17 longitudinal studies (*n* = 15,877, mean follow-up 7.7 years) reported that for a 1 m/s increase in cfPWV, the risk of future cardiovascular events, cardiovascular mortality, and all-cause mortality increased by 14, 15, and 15%, respectively (43). Our group has reported that uninterrupted prolonged sitting acutely increases cfPWV by 0.2–0.4 m/s (11–14). These changes may seem small in relation to a 1 m/s clinical cut point, but that cut-point is for chronic and not acute changes in cfPWV. The chronic changes in cfPWV are likely driven predominantly by changes in vessel structural, whereas the acute changes in cfPWV are mostly likely driven vessel function. In support, our group and others have reported that acute decreases in endothelial function are associated with increased arterial stiffness (20–22). The importance of these acute—and likely regular—SB-induced increases in cfPWV warrants further research. Additionally, further research is warranted to understand the pathophysiological implications of the 62% reduction if cfPWV we have reported when participants stand 30-min once/h decreased cfPWV by 62% vs. sitting only (11).

## Methodological Considerations

**Figure 6:**
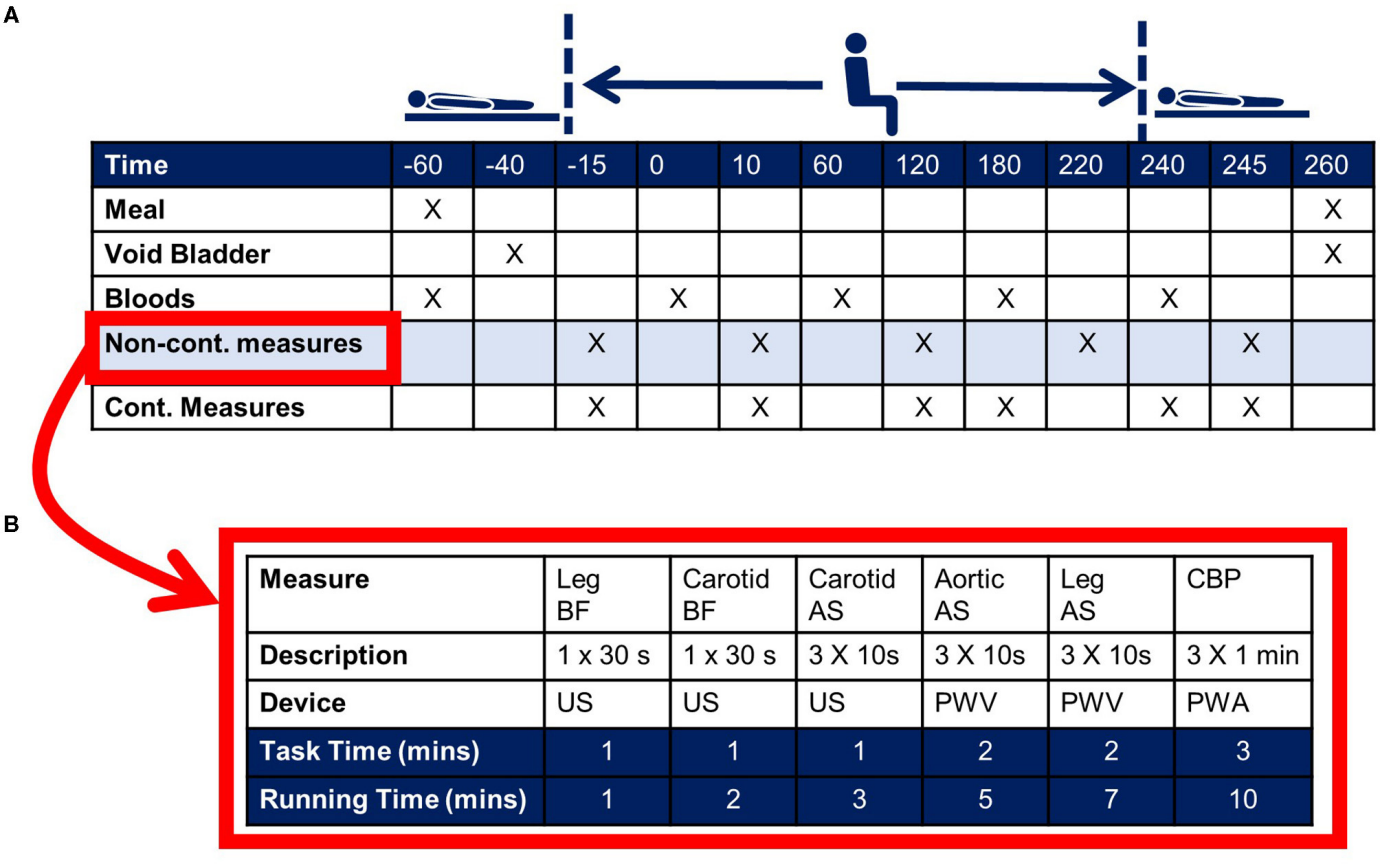
Example experimental protocol for an acute sitting study. **(A)** Indicates the time for a 3-h sitting study. Upon arrival to the laboratory the subject consumes a standard small meal/snack and venous blood is collected. The subject then rests quietly in the supine posture for 20-min prior to passive transfer to the seated posture. At the end of the 3-h sitting bout the subject is transferred back to the supine posture. Primary outcomes are collected in the supine posture. The cross marks indicate the measurement timepoints. **(B)** Indicates example non-continuous outcomes, including ultrasound (US)-derived leg blood flow (BF) and carotid arterial stiffness (AS), pulse wave velocity (PWV)-derived aortic (carotid-femoral)- and leg (thigh-ankle)-AS, and pulse wave analysis (PWA)-derived central blood pressure (CBP).

An example timeline for an acute sitting study is shown in Figure 6. This is for a single-day, uninterrupted sitting study design. Whether or not a single day is sufficient is dependent on the research question, the proposed mechanism(s), and the primary outcome. If a multiple-day protocol is required, consideration should be given to adherence to protocol, including the ability to measure adherence (i.e., *via* use of accelerometry). In the Figure 6 example the primary outcome, cfPWV, is part of the non-continuous battery of assessments (Figure 6B). These measurements are ordered with consideration to the chance of one measurement confounding the next. Continuous measurements may include blood pressure *via* volume-clamp plethysmography and heart rate *via* electrocardiogram. In this example, the non-continuous measurements are made pre- and post-sitting in the supine position, as well as each hour during the sitting bout. Though in accordance with recommendations for cfPWV assessments, hypothesis testing is based on measurements made in the supine position (44, 45). Measurements made in the seated position are used for monitoring trends, though repeated testing should be carefully considered as the measurement itself may confound the study outcomes (discussed further below).

The timeline shown in Figure 6 indicates one condition, uninterrupted sitting. However, it is highly likely that a researcher will want to compare this uninterrupted sitting condition to another condition, such as a strategy to interrupt sitting. For this scenario a decision has to be made as to whether a parallel group RCT design or cross-over study design is used. For practical reasons an RCT may not be feasible, as such designs require large sample sizes, and it is likely that—considering the time requirement—a given laboratory can test only one subject per day. For this reason, cross-over trials have commonly been used in the literature (11–14). This design permits each subject to serve as their own control, thereby eliminating between-subject variation as a statistical consideration and substantially increasing statistical efficiency. However, as will be discussed in Internal Validity the potential for a carry-over effect needs to be factored into the time between testing sessions. For both types of study design, consideration also needs to be given to treatment randomization. Randomizing subjects to control and treatment groups ensures that allocation to exposure is not determined by a third variable, which itself may influence the outcome. Ideally, consideration is given to both random allocation and random sampling, where random allocation is used to increase internal validity (see section Internal Validity) and random sampling increases generalizability (see section External Validity) to the population of interest. For acute sitting-based studies it is difficult, if not impossible, to blind the subject and research technician to the order of testing. However, the subjects and researcher technicians can be blinded to the condition until each day of testing, and the statistician and any technicians processing outcome data should be blinded.

Cross-over designs pose some additional statistical challenges. One challenge is loss to follow-up, and an additional challenge is accounting for confounders. With respect to loss to follow-up, this can occur when a given subject does not complete all testing days. A confounder is a variable that influences both the dependent variable and independent variable, causing a spurious association. For example, mean arterial pressure (MAP) is an important determinant of PWV (45). A change in MAP during and between each experiment testing day may confound the relationship between the sitting condition(s) and PWV. Collectively, these examples illustrate that the statistical strategy should be able to cope with missing data as well as time varying covariates. For these reasons mixed models have gained popularity.

While the statistical model is important, careful consideration should also be given to clinical inference. Clinical inference refers to whether a “significant” result is clinically beneficial or harmful. A *p*-value provides insight into whether or not the data are statistically significant within the context of the target population, i.e., the probability that the observed magnitude of difference for the sample is different than that of the intended target population, but does not provide information about clinical significance, i.e., whether the findings are likely to be meaningful with regards to physiology or pathophysiology (46). The practice of including statistical and clinical significance allows scientists to better infer the clinical and practical viability of study findings, which in turn will assist in translating the findings.

The magnitude of a clinically significant difference should be considered when making the study power calculation, i.e., the minimum sample size required to detect a true effect. Additional considerations include the importance of type I error (alpha level), which refers to incorrectly rejecting the null hypothesis, and type II errors (beta level), which refers to incorrectly accepting the null hypothesis. Lastly, the precision of the instrument selected to measure the primary outcome should be considered. This precision should be specific to the laboratory setting and observer, and specific to the testing conditions—particularly if measurements are made under non-standard conditions. For example, if FMD and PWV are measured in the seated posture, the precision estimates determined in the supine posture may not be appropriate. Additionally, as will be discussed in the next section, when measurements are made in the seated posture the device assumptions may be violated and measurement accuracy may be reduced.

## Internal Validity

The term “validity” is most commonly used in the context of the accuracy of a measurement tool, e.g., does a particular PWV device truly reflect the arterial stiffness of the segment of interest. However, validity also has a related meaning when it comes to the design of a study. There are two classes of threat to the validity of a study—internal validity and external validity. In the next section, we will discuss external validity. Here we focus on internal validity, which is concerned with the rigor (degree of control) of the study design. Controlling for confounding variables minimizes the potential for an alternative explanation for treatment effects and provides more confidence that any effects are due to the independent variable. For example, a study with high internal validity will control for the factors that may confound the true effect of acute prolonged sitting (independent) on the cardiovascular system (dependent variable).

The first set of threats to internal validity pertain to population. First, there is the threat of *regression to the mean*, which occurs when subjects have been selected on the basis of extreme scores, e.g., low and/or high FMD scores. With repeated testing, extreme (low and high) scores in a distribution tend to move closer to the mean, i.e., they regress (47). The second threat is treatment allocation. There should be an equal chance of allocation to each experimental condition, which can be controlled using *randomization*. In the case of cross-over trials, the order of conditions should be randomized. This process will help to control the confounding impact of the third threat, the “carry-over effect,” i.e., treatment one influencing the outcome for treatment two. However, while condition randomization will help to minimize this threat, attention also needs to be given to the minimum wash-out period to reduce any carry-over effect. Depending on the intervention conditions, it is likely that any carry-over effects can be “washed-out” with a minimal number of days. Third, consideration should be given to the *control group*. Control groups strongly affect inferences drawn from a study by ensuring that the interventional effects are beyond normal variation. In the case of a parallel group design, the control group should be appropriately matched to the experimental group(s). For cross-over designs the primary consideration is the *control condition*. That is, what is the appropriate control condition when investigating the effects of acute prolonged sitting on the cardiovascular system? Fourth, consideration needs to be given to *drop-out*, especially if those who drop-out from a study are different from those who remain in the study. This is a particular concern if a parallel group design is used, as drop-out may result in the groups no longer being matched.

The second set of threats pertain to extraneous factors that may confound the relationship between the variables of interest. These threats can be controlled through the appropriate employment of pre-assessment guidelines, and consideration to the environmental conditions during-assessment. Since vascular function is influenced by circadian and diurnal variation, it is typical to control experimental time of day with further consideration to the minimum number of awake hours prior to testing (48, 49). Additionally, changes in lifestyle behaviors can influence vascular function, including sleep, prior physical activity, and diet. While sleep has not traditionally been considered a component of pre-assessment control, arterial stiffness has been reported to increase following acute sleep deprivation (50). Therefore, in addition to establishing a minimum number of awake hours it may be beneficial to ask subjects to go to bed at the same time between visits. Dietary control may include providing a standardized meal for the night prior to testing and asking the subjects to report for testing having fasted. The fasted state is a typical requirement for vascular health testing as different macro- and micro-nutrients can greatly affect vascular function (51, 52). However, for studies incorporating long testing-day conditions, the fasted state may not be feasible. Additionally, having subjects fast may be unethical for certain population groups, such as type II diabetics who are at risk for hypoglycemia. For the aforementioned scenarios, careful consideration needs to be given to the timing and composition of the meal provided prior to testing. Ideally, to ensure consistency between testing sessions, the pre-assessment control factors should be actively monitored, including through the use of accelerometry to monitor physical activity and sleep, and diaries and continuous glucose monitors to monitor diet.

During-assessment, it is important to control environmental conditions, including ambient noise and temperature. Ambient noise may raise arousal, whilst core temperature and skin temperature have both been reported to affect vascular function (53). Fluid replacement should also be considered. Over a prolonged period, there will be insensible water loss, which could lead to a drop-in blood pressure, activate renin-angiotensin aldosterone system and confound the vascular outcome. As such, it important to estimate predicted water loss and put in place a strategy for water replacement. Lastly, considerations should be given to what subjects do and how they behave during the study. In terms of what they do, should they be permitted to engage in their typical desk work (e.g., homework assignment), or should they watch a non-stimulatory documentary to keep the subject awake while minimizing arousal? In terms of behavior, consideration needs to be given to bathroom breaks and how this is controlled for between study visits, as well as incidental movement (e.g., fidgeting) during the study. Ideally an accelerometer is worn on the ankle during the study so that movement can be used as a statistical covariate if needed.

The final set of threats pertains to instrumentation. To investigate the relationship between an independent and dependent variable, an instrument should be able to capture changes in the dependent variable with acceptable accuracy (validity) and precision (reliability). Moreover, it is important that the observer can report acceptable accuracy and precision in their laboratory setting, under the clearly articulated study conditions. For example, if measurements are made in the seated posture the accuracy and precision of measurements in said posture should be reported and used to determine statistical power. We have previously reported that both the seated posture and the non-fasted state decrease the precision of blood pressure and arterial wave reflection readings (54, 55). Additionally, we have reported that changing arm position relative to heart level impacted the generalized transfer function used for pulse wave analysis (56). Lastly, if measurements are made under non-standard conditions (i.e., with respect to guidelines, operating manual, and/or previous validation studies), it is important to understand the device assumptions and whether the non-standard conditions violate these assumptions. For example, pulse wave analysis is commonly used to measure augmentation index, an estimate of arterial wave reflection. One determinant of arterial wave reflection is arterial stiffness, and it may be expected that increased arterial stiffness during a bout of prolonged sitting would increase arterial wave reflection. However, we have reported a paradoxical decrease in augmentation index during prolonged sitting (12, 14). We rationalized that the decrease was a function of blood pooling in the lower-extremities, which would dampen the pressure wave.

### Flow-Mediated Dilation

As noted above, there has been interest on the effects of acute prolonged sitting on FMD as a measure of endothelial function. Our recent meta-analysis found that uninterrupted prolonged sitting decreased lower- but not upper-limb FMD (25). However, in interpreting these findings several considerations should be made, including accuracy, precision, the test assumptions, and measurement posture. Recall that accuracy refers to the closeness of a measurement to a known construct. The FMD test was originally validated by Celermajer et al. (57) for use in the brachial (upper-arm) artery, and shortly thereafter brachial artery FMD was validated against invasively assessed endothelial function in the coronary arteries (58). Subsequently, brachial FMD has been shown to be regulated by endothelium-derived molecules, most notably nitric oxide (NO) (59). As such, we have a good idea as to what brachial artery FMD is telling us (i.e., its validity). However, we know little about the accuracy of lower-limb FMD. Further, we know little about the precision of lower-limb FMD. One study has estimated the precision of popliteal artery FMD, reporting an ICC of 0.25 (60). To put this into context, we calculated the standard error of measurement from this aforementioned study to be 1.74%, meaning that for an average baseline FMD of 4.1%, a relative change exceeding 42% (i.e., 4.1–6.0%) would have to be observed to detect a true effect (61).

Test assumptions and measurement posture will be considered collectively. A major assumption is that the predominant stimuli regulating the FMD response (i.e., change in vessel tone) is the increase in shear stress during reactive hyperemia (38, 62). Shear stress is determined by red blood cells moving close to endothelial cells (see Figure 4B). As the fluid particles “travel” through the lumen, their velocity increases from zero at the vessel wall to a maximum value at the center of the vessel. This leads to the establishment of a gradient, which is defined as shear stress. Shear stress therefore acts at a tangent to the wall to create a frictional force at the surface of the endothelium. The endothelial cells are equipped with mechanosensors to detect this stress, enabling vascular tissues to respond with acute adjustments in vascular tone (59). This vasodilatory response reflects alterations in the rate of production of endothelial-derived mediators, including NO (59). NO performs a myriad of anti-atherogenic functions and is considered the most important molecule governing endothelial function and health (59). Therefore, it is assumed that FMD acts as a barometer of the bioavailability of these anti-atherosclerotic NO molecules. Ideally though, the vasodilatory response is adjusted for the shear stress stimulus. Fortunately, the duplex Doppler ultrasound equipment used for FMD can also estimate shear stress as the product of the shear rate and blood viscosity, where shear rate is estimated using an equation based on Poiseuille's law (38, 62). A major assumption of Poiseuille's law is that the velocity profile is parabolic, as depicted in Figure 4B. The velocity profile will generally not develop to a full parabola as a consequence of flow unsteadiness and short vessel entrance lengths, but the underestimation is likely not pronounced when resting in the supine position (63). However, in the seated posture, the shear stress profile in the lower-extremities (e.g., femoral or popliteal artery) may become turbulent during reactive hyperemia as a function of increased vessel tortuosity and hydrostatic pressure (64).

An alternative to taking FMD measurement in the seated posture is to take the measurements in the supine posture. Measurements in the supine posture are likely to be more accurate and precise, not only because of greater likelihood of capturing the true shear stress stimulus, but also because the measurements are less technically challenging. However, the caveat is that following the transition from the seated to supine posture, a short period of time, e.g., 10 min, will be required to ensure hemodynamic stability prior to FMD assessment. This may be particularly problematic for sitting interruption studies, as it will extend the time between the last interruption and the FMD assessment and, will likely mask the true interaction effect between prolonged sitting and the sitting interruption strategy. Conversely, by assessing FMD within 10 min of the final interruption (65), it could be argued that the subsequent elevation in shear stress as a consequence of the interruption will also mask the true effect of prolonged sitting. The optimal conditions for assessing the FMD response to prolonged sitting require careful consideration.

### Pulse-Wave Velocity

As with FMD assessments, accepted guidelines recommend measuring PWV in the supine posture (45). Additionally, as with any methodology, we should consider accuracy—what is the measurement reflecting? PWV, which is an estimate of arterial stiffness, is dependent on the functional and structural characteristics of a vessel (15, 22, 23). With prolonged sitting it is likely that changes in PWV reflect changes in vessel function. However, it should be considered that the device algorithm was validated in the supine posture (66), and that any changes in PWV in the seated posture may be confounded by changes in autonomic balance. During orthostasis there is a propensity for blood to pool in the sub-diaphragmatic venous system (67). To compensate for blood pooling, and attempt to ensure adequate venous return, the baroreflex response leads to increased lower extremity vascular tone (68). The increase in vascular tone leads to the fixed vessel mass being squeezed into a smaller space, which would increase PWV. To control for this potential confounding PWV should be adjusted for within-condition changes in MAP. However, it should be considered that the adjustment for MAP may not completely control for the confounding as it is a systemic rather than local measure. MAP does increase with vascular tone, but vascular tone is regulated by both autonomic and local factors, and that regulatory factors may differ by vessel segment.

Our group has compared PWV measures in the supine and seated posture (in review). The cfPWV was measured in the seated and supine postures prior to and following 3-h sitting in 18 young adults (mean age: 23 years, standard deviation [SD: 3] years). We found a posture effect, with higher cfPWV (β = 1.7 [SD: 0.14] m/s, *p* = 0.001) in the seated posture. However, no posture by time interaction effects were observed, with cfPWV changing similarly over time measures across postures. Across postures, we calculated an agreement of ICC = 0.77 (95%CI: 0.63–0.86) for cfPWV measurements made in supine and seated postures. In a separate study, carotid-radial (cr) PWV and carotid-ankle (ca) PWV were measured in the seated and supine postures prior to and following 7.5 h of sitting in 25 middle-aged adults (42 [SD: 12] years) (69). We again found a posture effect, with higher crPWV (β = 0.43 m/s, standard error [SE: 0.13], *p* = 0.001) and caPWV (β = 2.04 [SE: 0.17] m/s, *p* = 0.001) in the seated posture, but no posture by time interaction effect. Similarly, in 20 young adults (26 [SD: 8] years) we compared supine and seated caPWV and crPWV response to a caffeine perturbation, and found posture effects for caPWV (β = 0.55 m/s, *p* < 0.001) and caPWV (β = 3.59 m/s, *p* < 0.001), but no posture by time interaction effects (70). We also reported that crPWV (ICC: 0.77 vs. 0.64) and caPWV (ICC: 0.76 vs. 0.36) were more precise in the seated vs. supine posture (70), but that femoral-ankle PWV (faPWV) had acceptable precision in the supine (ICC: 0.83), but not seated posture (ICC = 0.29) (61). A poorer faPWV in a seated posture may be a consequence of orthostasis induced blood pooling compromising waveform detection. In summary, assessment posture does impact absolute PWV measures, but appears not to affect the observed response to prolonged sitting.

The standard measure of PWV is cfPWV, which represents the aorto-illiac pathway (15). This measure is particularly appealing because cfPWV is strongly associated with the risk of cardiovascular events (43), and international reference norms have been established for population, age, and risk factor strata (71). However, cfPWV assessments typically require applanation of the carotid artery. This can be technically challenging in certain populations, including persons who are obese and those with advanced carotid artery atherosclerosis (72), and accuracy may be compromised. Additionally, applanation of the carotid artery can result in a pressor reflex, which can become problematic with repeated measurements as the measurement itself may confound the study outcomes. An alternative PWV measure is heart-femoral PWV, here the “heart” portion can be determined from an electrocardiogram and/or a phonocardiogram. We recently found good (73) (*r* > 0.75) agreement between hfPWV and cfPWV (*r* = 0.83) in 4,133 older-aged (75 [SD: 5] years) adults) (74), and we also found that these measures responded similarly (*r* = 0.92) to an orthostatic challenge (modified tilt-table test), a perturbation known to impact aortic arterial stiffness (75). An additional alternative is brachial-femoral PWV (bfPWV), which can be obtained using oscillometric cuffs placed around the upper arm and upper thigh. Recently, we assessed bfPWV and cfPWV in the seated and supine postures prior to and following 3 h of sitting in 18 young adults (23 [SD: 3] years) and found acceptable overall agreement between the two measures (ICC: 0.74, 95%CI: 0.69–0.84) (in review).

## External Validity

External validity refers to whether your study results, obtained from a sample of the population of interest, can be generalized to the population at large. There is a reciprocal relationship between the external and internal validity, in that controlling for one often comes at the cost of the other. For example, with respect to acute sitting-based studies, the research setting is typically constrained to maximize internal validity. For example, it is common to control for population age, sex, time of day, diet, prior physical activity, and activities permitted during the study (e.g., use of the arms and legs). These factors are controlled so that the underlying causes of a phenomena can be established. However, in the real-world setting, the controlled variables may impact the relationship between prolonged sitting and the particular outcome of interest. Therefore, it is recommended that once a particular phenomenon has been established, subsequent consideration should be given to understanding whether said phenomena persists when extended to more presentative populations and settings. Two additional factors, which should be considered irrespective of the desired balance between internal validity and external validity are population sampling and population demographics.

As discussed above, the randomization component of an RCT is typically synonymous with the process through which subjects are allocated to groups. However, this process controls internal validity. To ensure external validity the population of interest should be randomly sampled (47). A number of sitting-based studies have used convenience sampling. That is, they have conducted the study using readily available higher education students. This is not surprising given the resources required to conduct acute sitting studies and the costs required for random sampling. However, with convenience sampling, individuals may not be representative of the larger population, thus, affecting both internal and external validity. To enable the reader to discern the generalizability of findings, the methods section should provide clear details about sampling and group allocations as well as inclusion and exclusion criteria, the results section should clearly articulate the sampling process and demographic characteristics, and the discussion section should contextualize who the study findings can be generalized to Slack and Draugalis (47).

Demographic considerations include age, race, and sex. Older adults (aged > 65 years) exhibit greater SB, with a recent meta-analysis indicating that older people are sedentary for 65–80% of their waking time (76). However, it is currently unknown whether the cardiovascular effects of acute prolonged sitting exposure differ in older compared to younger adults, and whether specific sitting interruption strategies are required to mitigate any unique negative cardiovascular effects. Similarly, we know that CVD mortality rate differs by race (77), but we do not know whether race interacts with the effects of repeated prolonged sitting exposure on the cardiovascular system. An additional consideration is sex. Men and women have different trajectories for the acquisition of CVD over the life course (78, 79). However, little is known about the effects of acute prolonged sitting exposure on the cardiovascular system in men vs. women (80). Further, because fluctuations in estrogen can affect cardiovascular measures, women are typically studied during the first 1–7 days of their menstrual cycle, or during the placebo week of contraceptive use. Controlling the stage at which women are tested increases internal validity, but at the expense of external validity. Although challenging experimentally, since larger sample sizes and/or more testing sessions will be required, it is important to understand the effects of repeated prolonged sitting exposure across the menstrual cycle so that we can better understand any unique sex effects.

To this point, we have discussed general population sampling and demographic issues, but attention should also be paid to clinical populations. For example, CVD is a major concern in people with Type II diabetes, and it is likely that this population will benefit from the hemodynamic effects of sitting interruption strategies. However, special attention needs to be given to glycemic control. Compared to fasting plasma glucose or glycated hemoglobin levels, hyperglycemic spikes are more strongly associated with CVD in this population (81), and the greatest hyperglycemic spikes occur following the consumption of each daily *ad libitum* meal (82). These spikes trigger oxidative stress, elevate inflammatory cytokines, reduce NO bioavailability, and induce endothelial dysfunction (32, 81, 83). As such, it may be particularly important to plan sitting interruption strategies around meal times in this group. Further, if glycemic control is the key regulatory mechanism, sitting interruption strategies should specifically target reductions in post-prandial glucose.

Recently, the WHO released the first global physical activity and SB guidelines for people living with disability (84). With respect to SB, similar to guidelines published for the general population (2, 3), the advice for reducing SB is vague and non-specific. When designing acute SB studies to address the gaps in knowledge, we need to consider that certain disabilities may preclude these individuals from interrupting SB through conventional means, and that these individuals may be at heightened risk for CMD. For example, one study using data from the Canadian Community Health Survey reported that the prevalence of heart disease (odds ratio [OR]: 2.7, 95%CI: 1.9–3.8) and stroke (OR: 3.7, 95%CI: 2.2–6.2) is much higher among those with a spinal cord injury (SCI) compared to those without (85). We have previously reported that the legs in in individuals with SCI are particularly susceptible to poor vascular health (86). A likely contributing factor is that persons with SCI may not be able to voluntarily contract skeletal muscle in their lower limbs, which limits hemodynamic stressors in this region, including decreased shear stress. To help lessen or reverse these regional abnormalities novel strategies will be required, of which regular sitting interruption may be a viable option. However, the strategies used to interrupt sitting behavior in able-bodied individuals are unlikely to be a suitable option. One potential alternative strategy is neuromuscular electrical stimulation to engage the lower-extremity musculature (87), and an additional is intermittent pneumatic compression therapy. The latter therapy, which is typically used to treat patients with peripheral arterial disease, can be used to raise lower extremity vascular shear stress. We recently reported that a single 60 min bout of intermittent pneumatic compression therapy acutely increased posterior tibial artery FMD by 31% (88).

## Ecological Validity

Ecological validity tells us whether or not our findings can be generalized to real-world or naturalistic settings. To put this concept into perspective, it is important to understand the mechanisms linking repeated prolonged sitting exposure to increased CVD risk, so that optimal sitting interruption prescription can be identified with respect to the FITT (frequency, intensity, time, and type) principle. However, the optimal FITT prescription will do little to lessen CVD risk if there is poor adherence to the prescription. As such there needs to be some compromise between what is considered to be the physiological optimal prescription vs. what people will actually do. For example, it is common practice to standardize the time of day when conducting an acute sitting-based study. This standardization is important to internal validity as circadian and diurnal variation can affect vascular outcomes (48, 49). In practice, this means our knowledge about the effects of prolonged sitting on the cardiovascular system are limited to effects that occur during the morning. We know little pertaining to whether these cardiovascular responses differ across the day, or whether sitting interruption strategies need to differ across the day to mitigate varying cardiovascular responses. Further, it is unclear if people will adhere to sitting interruption across the day, especially if these interruption strategies need to be varied.

A final consideration is the interaction with additional lifestyle behaviors. When people exercise, they tend to just exercise. This means that an optimal FITT prescription can be applied with limited consideration to other lifestyle behaviors. The same cannot be said for sitting behavior. People tend to engage in other behaviors while they are sitting, including consuming meals and performing stressful activities. This complicates both our physiological understanding and how we prescribe sitting interruption strategies. For example, we recently found that, in young healthy men (*n* = 13, 22 [SD: 2] years), combining prolonged sitting (3 h) with the consumption of a high-fat meal led to a greater increase in cfPWV (0.59 m/s, 95%CI: 0.29–0.89, Cohen's *d* = 1.12) when compared to a low-fat meal (0.14 m/s, 95%CI: 0.05–0.34, Cohen's *d* = 0.44) (in review). Additionally, in young, healthy adults (*n* = 18, 22.6 [SD: 3.1] years, 33% female) we investigated the effects of prolonged sitting (3 h) combined with the consumption of high- or low-glycemic index meal (26). Global PWV (a measure incorporating cf-, bf- and fa-PWV) increased by 0.29 m/s (95%CI: 0.14–0.45, *d* = 0.25), with a non-significant time effect. However, glucose variability was significantly correlated with bfPWV (*r* = 0.52, 95%CI: 0.19–0.71). In sum, these limited data indicate that special attention to concomitant lifestyle behaviors may be required when prescribing sitting interruption strategies.

## Translation

Ultimately, the goal of sitting-based research should be to inform policy that benefits the health of our societies. To provide context to this section, here we detail the considerations applied by the US Preventive Services Task Force (USPSF) when developing policy. The target audience of the USPSF is primary care clinicians, and its focus is conditions that cause a large burden of suffering to society and that also have available a potentially effective preventive service (89). In developing policy, the USPTF utilize a process similar to that used by many evidence-based groups, which is to systematically review the research literature and judge the strength of the evidence. The USPSTF assigns a grade ranging: from A (strongly recommended) to D (recommend against), or I (insufficient evidence) (89). This grade is assigned following three strata of admissible evidence, as follows: (i) individual studies (study design, internal validity); (ii) the linkage between each key question (aggregate internal validity, aggregate external validity, and coherence/consistency); and (iii) whether the evidence is adequate to determine the existence and magnitude of a causal connection between the preventive service and health outcomes. With respect to (i) individual studies, it is common for evidence-based groups to adjudicate the strength of individual studies based on a hierarchical grading system similar to that presented in Figure 2. This grading systems typically assigns the highest grade to RCTs (89). The USPSTF has extended their appraisal system to also consider RCTs cohort studies, case-control studies, and diagnostic accuracy studies (89). When appraising these studies, the USPSTF now separately rates the study design as well as internal and external validity (89). With respect to (ii) the linkage between each key question, the USPSTF considers aggregate internal- and external-validity, and the coherence of the body of evidence. Last, the USPSTF considers (iii) whether the evidence is adequate to determine the existence and magnitude of a causal connection between the preventive service and health outcomes. This includes assessing the magnitude of net benefit, in either words do the benefits outweigh potential harms? Additionally, in accordance with other agencies, the USPSTF considers the likelihood of a biologically plausible mechanism between cause and effect (89–91).

One such policy developed by the USPSTF is: “Healthful Diet and Physical Activity for Cardiovascular Disease Prevention in Adults With Cardiovascular Risk Factors: Behavioral Counseling” (92). The physical activity recommendation received a B grade—a recommendation that the service is routinely provided. Now consider that the first physical activity study was conducted in the 1950s (10), and since this time many well-controlled (internally-valid) RCTs have been conducted across numerous populations, and there is ample evidence to support net benefit to cardiovascular health (2). The sitting-based literature is currently void of well-designed cohort and RCTs that test mechanism-informed sitting interruption strategies. A major reason for this is a lack of true understanding of the effects of repeated exposure to uninterrupted prolonged sitting on the cardiovascular system. We need studies with high internal validity to assess mechanisms (biological plausibility) and to identify sitting interruption strategies that directly tackle the mechanisms of action. Establishing biological plausibility will facilitate the design of well-controlled RCTs that can be replicated across populations. Using the Pyramid of Clinical Evidence (Figure 2), we are currently *sitting* somewhere toward the bottom of the pyramid looking up. Accumulating the necessary evidence will take time and resources; physical activity did not receive such a lofty status over-night, but such status does shine a path.

## Conclusions

While leading public health agencies have begun to advocate a “reduction” in sitting behavior, these guidelines are typically vague and non-specific. The lack of specific guidelines for interrupting prolonged sitting bouts is attributable to the lack of available robust evidence to facilitate guideline development. To inform policy, well-designed RCTs are required to test the efficacy of specific and translatable sitting reduction strategies. To assist in the design of RCTs, this review postulated that several gaps in the literature first need to be filled, including the design of acute prolonged sitting-based studies that are internally valid and help to establish biological plausibility. The establishment of biological plausibility will assist the development of RCTs that target the mechanisms through which repeated sitting exposures stress the cardiovascular system. At present, we are some ways from developing specific sitting interruption guidelines. However, if, as a research community we consider the steps necessary to design well-controlled sitting exposure studies, while contemplating the criteria used to aid policy development, we can accelerate guideline development.

## Author Contributions

LS conceptualized the first draft. All authors contributed to revisions and approved for submission.

## Conflict of Interest

The authors declare that the research was conducted in the absence of any commercial or financial relationships that could be construed as a potential conflict of interest.

## Publisher's Note

All claims expressed in this article are solely those of the authors and do not necessarily represent those of their affiliated organizations, or those of the publisher, the editors and the reviewers. Any product that may be evaluated in this article, or claim that may be made by its manufacturer, is not guaranteed or endorsed by the publisher.
